# Colorectal Cancer in North-Eastern Iran: a retrospective, comparative study of early-onset and late-onset cases based on data from the Iranian hereditary colorectal cancer registry

**DOI:** 10.1186/s12885-021-09132-5

**Published:** 2022-01-08

**Authors:** Benyamin Hoseini, Zahra Rahmatinejad, Ladan Goshayeshi, Robert Bergquist, Amin Golabpour, Kamran Ghaffarzadegan, Fatemeh Rahmatinejad, Reza Darrudi, Saeid Eslami

**Affiliations:** 1 Pharmaceutical Research Center, Mashhad University of Medical Sciences, Mashhad, Iran; 2 Department of Medical Informatics, Faculty of Medicine, Mashhad University of Medical Sciences, Mashhad, Iran; 3 Department of Gastroenterology and Hepatology, Faculty of Medicine, Mashhad University of Medical Sciences, Mashhad, Iran; 4 Surgical Oncology Research Center, Mashhad University of Medical Sciences, Mashhad, Iran; 5 Formerly UNICEF/UNDP/World Bank/WHO Special Programme for Research and Training in Tropical Diseases (TDR), World Health Organization, Geneva, Switzerland; 6 Ingerod, SE-454 94 Brastad, Sweden; 7 School of Paramedical , Shahroud University of Medical Sciences, Shahroud, Iran; 8 Pathology Department, Education and Research Department, Razavi Hospital, Mashhad, Iran; 9 Department of Health Information Technology, Faculty of Paramedical Sciences, Mashhad University of Medical Sciences, Mashhad, Iran; 10 Department of Health Information Technology, Neyshabur University of Medical Sciences, Neyshabur, Iran; 11 Department of Medical Informatics, Academic Medical Center, University of Amsterdam, Amsterdam, the Netherlands

**Keywords:** Early-onset CRC, colon cancer, Colorectal cancer, Mismatch repair, cancer screening, cancer registry

## Abstract

**Background:**

The incidence rate of colorectal cancer (CRC) is increasing among patients below 50 years of age. The reason for this is unclear, but could have to do with the fact that indicative variables, such as tumour location, gender preference and genetic preponderance have not been followed up in a consistent mann er. The current study was primarily conducted to improve the hereditary CRC screening programme by assessing the demographic and clinicopathological characteristics of early-onset CRC compared to late-onset CRC in northeast Iran.

**Methods:**

This retrospective study, carried out over a three-year follow-up period (2014–2017), included 562 consecutive CRCs diagnosed in three Mashhad city hospital laboratories in north-eastern Iran. We applied comparative analysis of pathological and hereditary features together with information on the presence of mismatch repair (MMR) gene deficiency with respect to recovery versus mortality. Patients with mutations resulting in absence of the MMR gene *MLH1* protein product and normal BRAF status were considered to be at high risk of Lynch syndrome (LS). Analyses using R studio software were performed on early-onset CRC (*n* = 222) and late-onset CRC (*n* = 340), corresponding to patients ≤50 years of age and patients > 50 years.

**Results:**

From an age-of-onset point of view, the distribution between the genders differed with females showing a higher proportion of early-onset CRC than men (56% vs. 44%), while the late-onset CRC disparity was less pronounced (48% vs. 52%). The mean age of all participants was 55.6 ± 14.8 years, with 40.3 ± 7.3 years for early-onset CRC and 65.1 ± 9.3 years for late-onset CRC. With respect to anatomical tumour location (distal, rectal and proximal), the frequencies were 61, 28 and 11%, respectively, but the variation did not reach statistical significance. However, there was a dramatic difference with regard to the history of CRC in second-degree relatives between two age categories, with much higher numbers of family-related CRCs in the early-onset group. Expression of the *MLH1* and *PMS2* genes were significantly different between recovered and deceased, while this finding was not observed with regard to the *MSH6* and the *MSH2* genes. Mortality was significantly higher in those at high risk of LS.

**Conclusion:**

The variation of demographic, pathological and genetic characteristics between early-onset and late-onset CRC emphasizes the need for a well-defined algorithm to identify high-risk patients.

**Supplementary Information:**

The online version contains supplementary material available at 10.1186/s12885-021-09132-5.

## Introduction

Approximately 1.2 million people suffer from colorectal cancer (CRC), the third most deadly cancer worldwide [1]. In spite of overall decreasing CRC rates, particularly in patients older than 50 years [2], the trend is the opposite in younger patients [3, 4]. Between 1975 and 2010, the annual incidence rate per 100,000 of 20–49 year olds increased by 1.5% among males and 1.6% per year among females [3, 5]. Thus, although recent screening programmes by colonoscopy for CRC and other lower-intestine disorders have contributed to an overall decrease of the CRC incidence through the detection and elimination of precancerous polyps, this is not evident in those younger than 50 years [4, 6].

According to several studies, the incidence early-onset CRC (affecting those < 50 years of age) varies between different parts of the world with nearly 20% of the cases found in Asia including the Middle East (where the disease is not unusual in < 40-year olds) as compared to 2–8% reported in the U.S. [7, 8]. The mean age of early-onset CRC ranges between 37 and 47 years around the world [5, 7–18] and 41–69% of these cases are men. Compared to Western World (Europe and the U.S.), the incidence of CRC is currently very low in the older Iranian population, while young Iranians are showing a rising trend [19]. GLOBOCAN 2018 [20] tells us that the incidence of CRC could double in Iran before 2040.

The reason for the rising incidence and mortality of early-onset CRCs is unclear. Some authors suggest that the growing trend may be related to changing lifestyles, with an increasingly common type of patients characterized by overweight as evidenced by > 25 body mass index (BMI) and low physical activity, often also being current smokers, non-aspirin users and (pre) diabetics [7, 21–25]. A meta-analysis indicates that a CRC history in a first-degree relative (FDR), hyperlipidaemia, obesity and alcohol consumption are significant risk factors for early-onset CRC, while smoking, hypertension, metabolic syndrome, ulcerative colitis, chronic kidney disease, certain dietary factors, sedentary behaviour and occupational exposure to organic dusts can also be potential risk factors [26]. Biologically, CRC in young patients may be different from that seen in patients above 50 years of age. Previous studies have shown that CRC is mainly left-sided in young patients [12, 13] and particularly common in distal colon and rectum [12, 13, 27]. In addition, advanced-stage CRC with atypical histology has become more likely in younger patients, and they need more aggressive therapy compared to older individuals [28]. Current studies show that 94% of all early-onset CRCs are discovered and diagnosed after presenting with symptoms – the most predominant ones being abdominal/rectal pain and bleeding – something that indicates advanced stage with poor outcome [19, 29].

Considering the growing trend of this disease in younger patients, we need to integrate knowledge of early-onset CRC characteristics and differences to develop more precise and individualized screening and treatment strategies. So far, differences between early-onset and late-onset CRC have been investigated in various populations and ethnics to gain a better understanding of this upcoming world-wide health issue [30, 31]. While the pathogenesis of the former has been widely studied in the context of either hereditary syndromes or sporadic cases in the Western World [32–36], epidemiological data and pathogenesis of this type of cancer are generally lacking in countries in the Asia [22]. Moreover, there are considerable diversities regarding tumour location, gender preference and survival [31, 37–40], which need to be specified by region. To our knowledge, no study in Iran has addressed the characteristics of early-onset CRC with regard to screening and treatment strategies.

Some types of CRC are caused by a genetically inherited, autosomal disorder called the Lynch syndrome (LS) that increases the risk of many types of cancer, particularly CRC [18, 41–43]. The disorder is diagnosed by molecular or immunohistochemistry (IHC) testing in patients with mutations in one of four mismatch repair (MMR) genes designated *MLH1, PMS2, MSH6* and *MSH2*. Hereditary CRC is a priority of the Iranian Hereditary Colorectal Cancer Registry (IHCCR) that aims to detect, register and follow these patients. So far, identification of CRCs and colorectal adenomas at high risk of developing into hereditary CRC is recommended [18, 41, 42], but this may not be enough. The current study was conducted to assess the demographic, genetic and clinicopathological characteristics of early-onset CRC compared to late-onset CRC in Iran, specifically to improve screening for hereditary CRC.

## Materials and methods

### Setting and participants

**Fig. 1:**
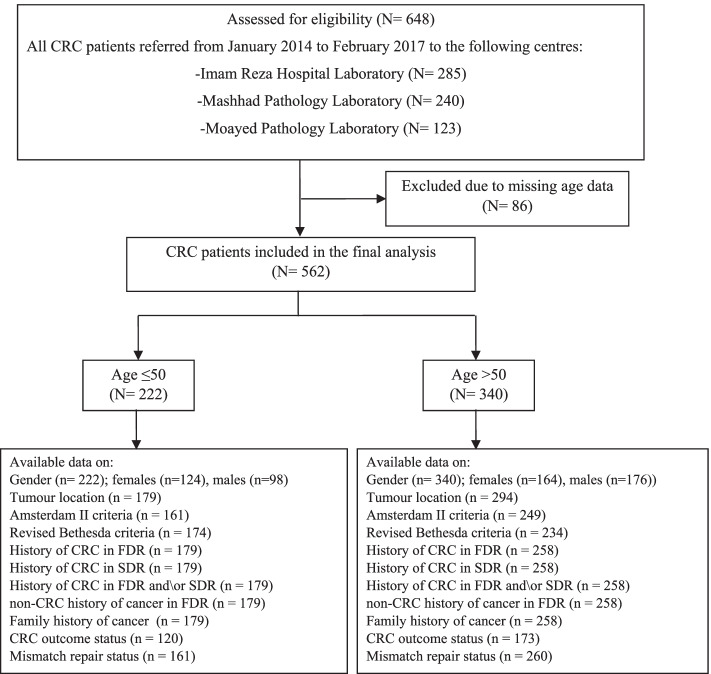
Schematic diagram of the inclusion/exclusion process. CRC = colorectal cancer; FDR = first degree relative; SDR = second degree relative

We approached the problem through the retrospective study of a cohort of consecutive CRCs between April 2014 and February 2017 in Mashhad City in north-eastern Iran. All patient information was obtained from three referral centres that included Imam Reza Hospital Laboratory, Mashhad Pathology Laboratory and Moayed Pathology Laboratory. Data on individuals without firm age information were discarded from the study, unless we were able to contact them and confirm how old they were at the time of diagnosis. With respect to other missing data, we considered each available item for each category and included also some variables with missing data as shown in Fig. 1 that illustrates the whole inclusion/exclusion process. Because of the variable availability of variables we ended up with different numbers of patients in the different categories. CRC cases at high risk of LS were included in IHCCR for later follow-up and genetic evaluation.

### Eligibility criteria

All CRCs consecutively registered in the databases of the three referral centres were eligible for inclusion. Cases with missing age data and/or clinically detected polyposis (> 10 polyps) were excluded. In case of unavailability of the surgical pathology slides used for IHC screening, the colonoscopy biopsy blocks were used instead. However, if both pathology slides and biopsy blocks were unavailable, the cases were excluded.

### MMR proteins immunohistochemistry

The diagnosis of MMR deficiency relies on the demonstration of absence of one or more gene protein products. We performed IHC for the four most common MMR proteins using the standard procedure based on primary monoclonal antibodies from Vitro SA, Master Diagnostica, Spain (https://www.vitro.bio/inicio), i.e. clone BS29 for MLH1, clone FE11 for MSH2, clone EP49 for MSH6 and clone BS29 for PMS2. Deparaffinized, rehydrated and heat-induced 4-mm tissue sections were used for epitope retrieval with the reaction visualized with Novolink polymer (Leica Company, Wetzlar, Germany). The slides were counterstained with haematoxylin and eosin. Two expert pathologists evaluated the results independently and blindly. All patients received therapy by surgery and chemotherapy according to CRC stage and oncologist opinion.

#### Outcome measures and variables

The Amsterdam II [44] and the revised Bethesda [45] guidelines were followed during the study to improve identification of individuals likely to have LS and therefore being at increased risk of developing CRC [18, 41, 42]. The screening for CRC in patients at high risk of LS focused on the protein products of the four MMR genes *MLH1, MSH2, MSH6* and *PMS2* as identified by absence of a specific IHC screening. To exclude sporadic CRCs with acquired promoter hypermethylation, tumours without IHC staining for *MLH1* were tested with reference to a mutation of the B-Raf proto-oncogene serine/threonine kinase (BRAF) gene that could result in a valine-to-glutamate change at residue no. 600 (V600E) [16].

The secondary outcome measure was CRC outcome, which was based on the conclusion at the hospital (death or recovery and discharge). We compared available data between deceased and recovered patients with respect to gender, anatomical tumour location, status of the DNA MMR genes according to the Amsterdam II [44] and the revised Bethesda [45] criteria, CRC history in first and second degree relatives (FDR and SDR), as well as absence of CRC in the family history of cancer. Also, we compared these variables between early-onset and late-onset CRC.

### Statistical analyses

The Chi-square test and the Fisher exact test were employed to identify any statistically significant differences in baseline characteristics in relation to age. The data were presented as percentage frequency of categorical variables and with the mean standard deviation (SD) for continuous variables. *P*-values < 0.05 were considered statistically significant. All analyses were performed using R studio software (https://rstudio.com/).

## Results

### Demographic characteristics

As seen in Fig. 1, a total of 562 CRC cases were included in the study. The patients were divided into two categories depending on when the diagnosis was made: early-onset CRC (*n* = 222) with patients ≤50 years of age and late-onset CRC (*n* = 340) with those older than that. The participants’ ages ranged from 20 to 90 years with a mean age of 55.63 ± 14.8 years. In the early-onset CRC group, the mean age was 40.34 ± 7.3 years, while it was 65.11 ± 9.3 years in the late-onset group. The overall gender distribution was close to equal (51% females vs. 49% males). The outcomes in relation to the different characteristics studied for the two age categories are shown in Table 1; however, because this was a retrospective study, we were unable to collect all data for all variables investigated, which is given for each entry in the Table.

**Table 1:** Baseline characteristics of the study participants

Characteristic	Early-onset CRC (%)	Late-onset CRC (%)	***P***-value
Gender (*n* = 562)			
Female (*n* = 288)	124 (55.9)	164 (48.2)	0.046^c^
Male (*n* = 274)	98 (44.1)	176 (51.8)	
Tumour location (*n* = 473)			
Proximal (*n* = 53)	16 (8.9)	37 (12.6)	
Distal (*n* = 287)	111 (62.0)	176 (59.9)	0.47^c^
Rectum (*n* = 133)	52 (29.1)	81 (27.6)	
Amsterdam II (*n* = 410)			
Criteria absent (*n* = 395)	151 (93.8)	244 (98.0)	**0.03**^c^
Criteria present (*n* = 15)	10 (6.2)	5 (2.0)	
Revised Bethesda (*n* = 408)			
Criteria absent (*n* = 212)	14 (8.0)	198 (84.6)	**0.001**^c^
Criteria present (*n* = 196)	160 (92.0)	36 (15.4)	
History of CRC in FDR (*n* = 437)			
No (*n* = 395)	162 (90.5)	235 (90.3)	0.9^c^
Yes (*n* = 42)	17 (9.5)	25 (9.7)	
History of CRC in SDR (*n* = 437)			
No (*n* = 399)	154 (86.0)	245 (95.0)	**0.001**^c^
Yes (*n* = 38)	25 (14.0)	13 (5.0)	
History of CRC in FDR or SDR (*n* = 437)			
No (*n* = 362)	141 (78.8)	221 (85.7)	0.06^c^
Yes (*n* = 75)	38 (21.2)	37 (14.3)	
History of CRC in FDR and SDR (*n* = 437)			
No (*n* = 432)	175 (97.8)	257 (99.6)	0.16^d^
Yes (*n* = 5)	4 (2.2)	1 (0.4)	
non-CRC history of cancer in FDR (*n* = 437)			
No (*n* = 374)	155 (86.6)	219 (84.9)	0.6^c^
Yes (*n* = 63)	24 (13.4)	39 (15.1)	
Family history of cancer (*n* = 437)			
Absent (*n* = 291)	114 (63.7)	177 (68.6)	0.3^c^
Present (*n* = 146)	65 (36.3)	81 (31.4)	
CRC outcome group (*n* = 293)			
Recovered and discharged (*n* = 234)	103 (85.8)	131 (75.7)	**0.03**^c^
Deceased at the hospital (*n* = 59)	17 (14.2)	42 (24.3)	
Mismatch repair status (*n* = 421)			
Proficient (*n* = 380)	143 (88.8)	237 (91.1)	0.39^d^
Deficient (*n* = 41)	18 (11.2)	23 (8.9)	
At high risk for LS^a^ (*n* = 421)			
No (*n* = 385)	143 (88.8)	242 (93.1)	
Yes (*n* = 36)	18 (11.2)	18 (6.9)	0.14^d^
MMR-deficiency^b^ (*n* = 41 (	18 (43.9)	23 (56.1)	
*MLH1* (*n* = 27)	13 (48.1)	14 (51.9)	
*PMS2* (*n* = 30)	13 (43.3)	17 (56.7)	0.3^d^
*MSH2* (*n* = 9)	4 (44.4)	5 (55.6)	
*MSH6* (*n* = 10)	5 (50.0)	5 (50.0)	

*CRC* colorectal cancer, *FDR* first degree relative, *SDR* second degree relative, *LS* Lynch syndrome, *MMR* mismatch repair

^a^ CRCs with absent MMR proteins, and normal BRAF status (if *MLH1* was absent)

^b^ Some MMR gene deficiencies included more than one protein, which explains why the sum exceeds 41

^c^ Analysis by Chi-square test

^d^ Analysis by Fisher’s exact test

### Clinicopathological characteristics

With respect to the early-onset CRC and late-onset CRC categories, there was a statistically significant difference between the two age categories at the *p* = 0.03 level. Interestingly, women made up the majority of early-onset CRCs compared to the late-onset ones. The anatomical tumour location varied considerably between the two types of CRC, but did not reach statistical significance, although it was evident that distal colorectal tumours were in majority (61%), particularly in early-onset CRC (62%). The Amsterdam II criteria were mostly present among the early-onset CRCs, and 42% of them had MMR-deficiencies as well. The Revised Bethesda criteria reinforced this impression and at a stronger level of statistical significance (Table 1). About 83% of patients had no history of CRC, neither in FDR nor in SDR, and around 14% of 437 CRCs had non-CRC history of cancer in FDR, the most common being gastric cancer, breast cancer and lung cancer. Generally, there was no statistically significant difference between the early-onset CRC and late-onset CRC groups with respect to history of CRC in FDR; however there was a drastic difference with regard to history of CRC in SDR. Among 437 CRCs, about 33% had reported family history of cancer (Table 1).

### IHC screening

With regard to IHC investigation, we had only access to 41 patients, several of whom had more than one kind of MMR-deficiency (dMMR). Although the percentage of MMR-deficiency in the early-onset CRC group was greater than that in the late-onset one, this difference was not significant. Twenty-eight cases with loss of *MLH1* underwent testing for the BRAF mutation, 5 of whom were recognized as positive for the BRAF mutation and excluded as sporadic CRC. Finally, 36 of 41 dMMR CRCs with mean age of 51.9 ± 14.1 years were detected as being at high risk of LS (Table 1).

### CRC outcome

Of 293 CRCs with available outcome, 20% passed away, a fact that was significantly more common among the late-onset CRCs than the early-onset CRC ones (Table 1). Several features between the deceased and the recovered groups, including deficient expression of the *MSH6* and *MSH2* genes; the Amsterdam- II/revised Bethesda criteria, as well as the tumour location, did not reach statistical significance. However, age, presence of deficient *MLH1* and *PMS2* genes as well as risk for LS stood out as high-impact variables (Table 2).

**Table 2:** Various features in the group of patients studied with respect to CRC outcome

CRC outcome Variable	Recovered = 234 Number (%)	Deceased = 59 Number (%)	Total = 293 Number (%)	***P***-value
Continuous age - mean (SD)	53.58 (14.11)	59.25 (16.88)	54.72 (14.86)	**0.009**
Age range (years)				
20–29	8 (3.4)	3 (5.1)	11 (3.8)	
30–39	31 (13.2)	6 (10.2)	37 (12.6)	
40–49	55 (23.5)	8 (13.6)	63 (21.5)	**0.03**^d^
50–59	57 (24.4)	11 (18.6)	68 (23.2)	
60–69	40 (17.1)	10 (16.9)	50 (17.1)	
70–79	35 (15.0)	13 (22.0)	48 (16.4)	
≥ 80	8 (3.4)	8 (13.6)	16 (5.5)	
Gender				
Female	156 (49.0)	29 (49.0)	185 (49.0)	0.9^c^
Male	159 (51.0)	30 (51.0)	189 (51.0)	
Amsterdam II				
Criteria absent	196 (95.1)	53 (96.3)	249 (95.0)	0.7^c^
Criteria present	10 (4.9)	2 (3.7)	12 (5.0)	
Tumour location				
Proximal	30 (14.2)	6 (10.9)	36 (13.5)	0.2^c^
Distal	126 (59.4)	28 (50.9)	154 (57.7)	
Rectum	56 (26.4)	21 (38.2)	77 (28.8)	
Mismatch repair status				
Proficient	143 (92.0)	31 (77.5)	174 (89.0)	**0.02**^c^
Deficient	13 (8.0)	9 (22.5)	22 (11.0)	
At high risk for LS^a^				
No	145 (93.0)	32 (80.0)	177 (90.0)	**0.03**^c^
Yes	11 (7.0)	8 (20.0)	19 (10.0)	
*MLH1*				
pMMR	147 (94.0)	34 (85.0)	181 (92.0)	**0.05**^c^
dMMR	9 (6.0)	6 (15.0)	15 (8.0)	
*PMS2*				
pMMR	145 (94.0)	33 (82.0)	178 (91.0)	**0.02**^c^
dMMR	10 (6.0)	7 (18.0)	17 (9.0)	
*MSH2*				
pMMR	152 (98.0)	38 (95.0)	190 (97.0)	0.27^c^
dMMR	3 (2.0)	2 (5.0)	5 (3.0)	
*MSH6*				
pMMR	153 (98.0)	38 (95.0)	191 (97.0)	0.27^c^
dMMR	3 (2.0)	2 (5.0)	5 (3.0)	

*CRC* colorectal cancer, *MMR* mismatch repair, *pMMR* mismatch repair proficiency, *dMMR* mismatch repair-deficiency, *LS* Lynch syndrome

^a^Defined as the CRC cases with absent MMR proteins and normal BRAF status (if *MLH1* was absent)

^c^ Analysis by Fisher’s exact test

^d^ Analysis by Chi-square test

## Discussion

Multiple studies have focused on clarifying the characterization of CRC based on the age of onset [5, 46]. However, to our knowledge, this is the first study in Iran to assess the demographic, clinicopathological and hereditary features in cases diagnosed before 50 years, the age generally used to separate early-onset from late-onset CRC. Although several features (Amsterdam-II, Revised Bethesda, tumour location, deficient expression of the *MSH6* and *MSH2* genes) did not reach statistical significance, others (LS risk and deficient expression of the *MLH1* and *PMS2* genes) differed dramatically with respect to CRC outcome. Most CRC cases in the late-onset CRC group and those diagnosed as being at high risk of LS died prematurely because of the cancer. In spite of this fact, our results support the understanding that early-onset CRC is in a long-term, rising trend, while the opposite is true for late-onset CRC. These trends are evident at regional as well as country levels, e.g., an Italian study has shown that the incidence rate of CRC in patients aged 20–49 years increased from 9.3 in 1957 to 13.7 in 2015, whereas the incidence rate of CRC in older patients has steadily declined [2]. Recent studies report incidence rates in CRC patients ≤50 years in India [47] and in the central region of Iran [22] at 39 and 25%, respectively. Furthermore, we found that more than 39% of our cases were first diagnosed with CRC when still under 50 years. We also found that the mean age for early-onset CRCs was 40 years and that the highest number of cases were in the final decade of the age range investigated (40–50 years of age), which is in accordance with other studies [9, 11, 16, 48]. However, this pattern is more pronounced in Asia than in the Western countries [38, 49–55].

In the last 10 years, a large number of research projects around the world have focused on early-onset CRC. The sample size of these studies ranged between 49 and 64,068 cases [5, 7–18, 48, 56–58] and the mean age of study subjects ranged between 37 and 47 years [5, 7–12, 14–18]. In contrast to our study, the frequency of early-onset CRC is generally reported to be higher in men than in women [5, 7, 10, 11, 13–17, 48], but various studies have yielded conflicting results [10, 28, 47, 59]. Without finding any reason for the gender difference, we noted that early-onset CRC predominantly affected women, whereas late-onset CRC mainly involved men, which was statistically significant.

With regard to localization, our analysis revealed that CRC was less frequent in the proximal colon, which is consistent with results by some authors in the Middle-East [15, 22, 57, 58]. However, the results of other studies performed in the Western World diverge [31, 60], which suggests that there may be differences with regard to the pathogenesis and aetiology of this kind of tumour between the Middle-East and the West. In accordance with our results, which showed an identical distribution of distal tumours in both CRC groups, there seems to be no significant difference with respect to age and tumour location in different age groups [28, 37, 61, 62], On the other hand, the results of multiple other studies are not in line with these findings [10, 47, 63, 64], so tumour localization remains a contended issue.

Although family history of cancer overall did not align with any of the two age categories studied at a statistical significant level, the presence of a positive history of CRC in SDR together with early-onset CRC was highly significant and the combination with either FDR or SDR did not reach significance. These observations confirm previous research results on this subject [10, 65].

It has been observed that tumour histology in younger patients often show poor differentiation with more aggressive growth compared to that seen in older patients [66, 67]. Although this is generally negative for recovery, the overall 5-year relative survival rate for patients under 50 years is on the whole not shorter than for patients above this age [37, 40, 68, 69]. Indeed, despite young patients often present with more advanced disease, it is not unusual for them to survive longer, a counterintuitive fact that is supported by the significantly higher mortality for late-onset CRCs in our study. On the other hand, this must be seen in connection with the concomitant life expectancy.

Multiple risk factors contribute to CRC development. This has been assessed in Iran previously [70–72] and among the various factors affecting CRC incidence, the main reason for early-onset CRC development might be the various germline mutations that have come to light in the last two decades [18, 73]. Our study, in addition to demographic and pathological characteristics, addressed the MMR status of early-onset CRCs. Although this study could not confirm germline MMR mutations in other ways than by IHC, the assessment of CRC cases at high risk of LS with respect to age and outcome illuminated the background to how genetic aspects may play a role in CRC development by finding that CRC in those at high risk of LS was more prevalent in the deceased group. With 83% sensitivity and 89% specificity of IHC testing for absence of expression of MMR proteins in this group, we are confident that there is a connection. Tumours in LS cases also demonstrate microsatellite instability (MSI) owing to loss of DNA MMR, and testing for this by the polymerase chain reaction (PCR), which amplifies a standard panel of DNA sequences containing nucleotide repeats, has a sensitivity and specificity of about 85 and 90%, respectively [43, 74]. Loss of the *MLH1* expression owing to hypermethylation of this protein is seen in about 15% of sporadic CRCs based on IHC screening [75]. On the other hand, cancers associated with the *MSH6* protein may be missed during MSI testing because this gene is preferentially involved in repair of mononucleotide repeats and mononucleotide markers are not included in all MSI panels [43, 76]. In spite of the better sensitivity and specificity of MSI testing, IHC is cheap and easily available, can be conducted using small biopsies and has the added value of assisting identification of the MMR protein(s) that may have caused the dMMR-related tumour.

### Strengths and limitations

The multicentre design with a relatively large number of patients followed over a period of 3 years is the main strength of the present study. In addition, the study examines thoroughly the various features in terms of age group and CRC outcome. Since we were not able to contact all CRC cases, the number of patients to deal with changed between the variables as can be seen in Table 1. This was the most challenging limitation, but what was available still allowed us to achieve acceptable power of the statistics used. We compared the characteristics of deceased and recovered CRC cases based on hospital mortality only for 293 consecutive CRCs. Thus, we could not perform survival analyses. However, this was not the aim of the study and will be addressed in future studies. Data, such as symptoms, socioeconomic status, BMI, past medical history, ethnicity, geographic and colonoscopy findings were not available for all cases and must be counted as another limitation as a complete dataset would have made a more detailed interpretation possible. Owing to lack of genetic testing in our setting, the study applied a strategy to identify CRC cases at high risk for LS under the circumstances and resources available. This made differentiation between true Lynch and Lynch-like cases difficult, but this limitation can be avoided in the future as genetic diagnosis is becoming available locally. Furthermore, due to lack of genetic evaluation, we aimed to detect polyposis clinically but no familial adenomatous (FAP) cases were found. The limitations mentioned impose a lack of generalizability of the findings and methods used to characterize early-onset CRCs, but we still think that the results will be useful for low- and middle-income countries especially in Middle East where their resources are as limited as in Iran.

## Conclusion

With up to 57% of the early-onset CRC cases at ages of 40 and 49, the mortality rate in this group was considerable. Furthermore, MMR deficiency and risk of LS in CRC patients was more common in the deceased group than among those who survived, but this difference was only significant with respect to the *MLH1* and *PMS2* genes. In our study, women were in majority for early-onset CRC, while the opposite was the case for late-onset CRC. The incidence of CRC with distal tumours was frequently higher than for other sites, but there was no difference between the early-onset and late-onset CRC cases. Taken together, these findings highlight the need for a well-defined algorithm assisting the identification of patients at risk for early-onset CRC.

## Supplementary Information

**Additional file 1.**

## Data Availability

The demographic and clinical datasets generated and/or analysed during the current study are available in the Harvard Dataverse repository, [10.7910/DVN/HHLMA1]. The molecular genetic dataset used and/or analysed during the current study are available from the corresponding author (Dr. Ladan Goshayeshi) on reasonable request.

## References

[1] Rawla P, Sunkara T, Barsouk A (2019). Epidemiology of colorectal cancer: incidence, mortality, survival, and risk factors. Przegl Gastroenterol.

[2] Russo A, Andreano A, Sartore-Bianchi A, Mauri G, Decarli A, Siena S (2019). Increased incidence of colon cancer among individuals younger than 50 years: a 17 years analysis from the cancer registry of the municipality of Milan. Italy Cancer Epidemiol.

[3] Bailey CE, Hu CY, You YN, Bednarski BK, Rodriguez-Bigas MA, Skibber JM (2015). Increasing disparities in the age-related incidences of colon and rectal cancers in the United States, 1975-2010. JAMA Surg.

[4] Siegel RL, Jemal A, Ward EM (2009). Increase in incidence of colorectal cancer among young men and women in the United States. Cancer Epidemiol Prev Biomark.

[5] Arriba M, Sánchez C, Vivas A, Nutu O, Rueda D, Tapial S (2019). Intermediate-onset colorectal cancer: a clinical and familial boundary between both early and late-onset colorectal cancer. PLoS One.

[6] Jalili-Nik M, Soltani A, Moussavi S, Ghayour-Mobarhan M, Ferns GA, Hassanian SM (2018). Current status and future prospective of Curcumin as a potential therapeutic agent in the treatment of colorectal cancer. J Cell Physiol.

[7] Montazeri M, Hoseini B, Firouraghi N, Kiani F, Raouf-Mobini H, Biabangard A, et al. Spatio-temporal mapping of breast and prostate cancers in South Iran from 2014 to 2017. BMC cancer. 2020;20(1):1170. Epub 2020/12/02. 10.1186/s12885-020-07674-8. PubMed PMID: 33256668; PubMed Central PMCID: PMCPMC7708260. 10.1186/s12885-020-07674-8 PMC7708260 33256668

[8] Glover M, Mansoor E, Panhwar M, Parasa S, Cooper GS (2019). Epidemiology of colorectal Cancer in average risk adults 20–39 years of age: a population-based National Study. Dig Dis Sci.

[9] You YN, Xing Y, Feig BW, Chang GJ, Cormier JN (2012). Young-onset colorectal cancer: is it time to pay attention?. Arch Intern Med.

[10] Myers EA, Feingold DL, Forde KA, Arnell T, Jang JH, Whelan RL (2013). Colorectal cancer in patients under 50 years of age: a retrospective analysis of two institutions' experience. World J Gastroenterol.

[11] Perrott S, Laurie K, Laws K, Johnes A, Miedzybrodzka Z, Samuel L (2020). Young-onset colorectal cancer in the north east of Scotland: survival, clinico-pathological features and genetics. BMC Cancer.

[12] Zhunussova G, Afonin G, Abdikerim S, Jumanov A, Perfilyeva A, Kaidarova D (2019). Mutation spectrum of cancer-associated genes in patients with early onset of colorectal cancer. Front Oncol.

[13] Ferlay J, Soerjomataram I, Dikshit R, Eser S, Mathers C, Rebelo M (2015). Cancer incidence and mortality worldwide: sources, methods and major patterns in GLOBOCAN 2012. Int J Cancer.

[14] Rashid MU, Naeemi H, Muhammad N, Loya A, Lubiński J, Jakubowska A (2019). Prevalence and spectrum of MLH1, MSH2, and MSH6 pathogenic germline variants in Pakistani colorectal cancer patients. Hereditary Cancer Clin Pract.

[15] Maraqa B, Al-Shbool G, Abu-Shawer O, Souleiman M, Alshakhatreh O, Al-Omari A (2020). Frequency of mismatch repair protein (MMRP) deficiency among young jordanians diagnosed with Colorectal Carcinoma (CRC). Gastroenterol Res Pract.

[16] Ashktorab H, Brim H, Al-Riyami M, Date A, Al-Mawaly K, Kashoub M (2008). Sporadic colon cancer: mismatch repair immunohistochemistry and microsatellite instability in Omani subjects. Dig Dis Sci.

[17] Gausman V, Dornblaser D, Anand S, Hayes RB, O'Connell K, Du M (2020). Risk Factors Associated With Early-Onset Colorectal Cancer. Clin Gastroenterol Hepatol.

[18] Goshayeshi L, Ghaffarzadegan K, Khooei A, Esmaeilzadeh A, Rahmani Khorram M, Mosannen Mozaffari H (2018). Prevalence and clinicopathological characteristics of mismatch repair-deficient colorectal carcinoma in early onset cases as compared with late-onset cases: a retrospective cross-sectional study in Northeastern Iran. BMJ Open.

[19] Malekzadeh R, Bishehsari F, Mahdavinia M, Ansari R. Epidemiology and molecular genetics of colorectal cancer in iran: a review. Archives of Iranian medicine. 2009;12(2):161–9. Epub 2009/03/03. PubMed PMID: 19249887. 19249887

[20] Bray F, Ferlay J, Soerjomataram I, Siegel RL, Torre LA, Jemal A (2018). Global cancer statistics 2018: GLOBOCAN estimates of incidence and mortality worldwide for 36 cancers in 185 countries. CA Cancer J Clin.

[21] Sudarshan V, Hussain N, Gahine R, Mourya J (2013). Colorectal cancer in young adults in a tertiary care hospital in Chhattisgarh, Raipur. Indian J Cancer.

[22] Zeinalian M, Hashemzadeh-Chaleshtori M, Akbarpour MJ, Emami MH (2015). Epidemioclinical feature of early-onset colorectal Cancer at-risk for Lynch syndrome in Central Iran. Asian Pac J Cancer Prev: APJCP.

[23] Esmaeilzadeh A, Goshayeshi L, Bergquist R, Jarahi L, Khooei A, Fazeli A (2021). Characteristics of gastric precancerous conditions and *Helicobacter pylori* infection among dyspeptic patients in north-eastern Iran: is endoscopic biopsy and histopathological assessment necessary?. BMC Cancer.

[24] Goshayeshi L, Hoseini B, Yousefli Z, Khooie A, Etminani K, Esmaeilzadeh A (2017). Predictive model for survival in patients with gastric cancer. Electron Physician.

[25] Azizi A, Aboutorabi R, Mazloum-Khorasani Z, Hoseini B, Tara M (2016). Diabetic personal health record: a Ssystematic review article. Iran J Public Health.

[26] O'Sullivan DE, Sutherland RL, Town S, Chow K, Fan J, Forbes N, et al. Risk factors for early-onset colorectal Cancer: a systematic review and Meta-analysis. Clin Gastroenterol Hepatol. 2021. 10.1016/j.cgh.2021.01.037 Epub 2021/02/02. PubMed PMID: 33524598. 10.1016/j.cgh.2021.01.037 33524598

[27] Ladan Goshayeshi, Mina Akbari Rad, Robert Bergquist, Abolghasem Allahyari, members of the MUMS Covid-19 Research Team, Benyamin Hoseini. Demographic and Clinical Characteristics of the Severe Covid-19 Infections: First Report from Mashhad University of Medical Sciences, Iran. medRxiv. 2020:2020.05.20.20108068. 10.1101/2020.05.20.20108068. 10.1186/s12879-021-06363-6 PMC8261035 34233638

[28] De Silva M, Fernando M, Fernando D (2000). Comparison of some clinical and histological features of colorectal carcinoma occurring in patients below and above 40 years. Ceylon Med J.

[29] Siegel RL, Sahar L, Robbins A, Jemal A (2015). Where can colorectal cancer screening interventions have the most impact?. Cancer Epidemiol Prev Biomark.

[30] Schellerer V, Croner R, Langheinrich M, Hohenberger W, Merkel S (2015). Colorectal carcinoma in young patients-is age a prognostic factor?. Zentralbl Chir.

[31] Perea J, Rueda D, Canal A, Rodríguez Y, Álvaro E, Osorio I (2014). Age at onset should be a major criterion for subclassification of colorectal cancer. J Mol Diagn.

[32] Popat S, Hubner R, Houlston RS (2005). Systematic review of microsatellite instability and colorectal cancer prognosis. J Clin Oncol.

[33] Chang DT, Pai RK, Rybicki LA, Dimaio MA, Limaye M, Jayachandran P (2012). Clinicopathologic and molecular features of sporadic early-onset colorectal adenocarcinoma: an adenocarcinoma with frequent signet ring cell differentiation, rectal and sigmoid involvement, and adverse morphologic features. Mod Pathol.

[34] Jandova J, Xu W, Nfonsam V (2016). Sporadic early-onset colon cancer expresses unique molecular features. J Surg Res.

[35] Pilozzi E, Lorenzon L, Lo Baido S, Ferri M, Duranti E, Fochetti F (2017). Left-sided early onset colorectal carcinomas: a sporadic neoplasm with aggressive behavior. Am J Surg.

[36] Stigliano V, Sanchez-Mete L, Martayan A, Anti M (2014). Early-onset colorectal cancer: a sporadic or inherited disease?. World J Gastroenterol.

[37] Schellerer V, Croner R, Langheinrich M, Hohenberger W, Merkel S (2015). Colorectal carcinoma in young patients - is age a prognostic factor?. Zentralbl Chir.

[38] Mahdavinia M, Bishehsari F, Ansari R, Norouzbeigi N, Khaleghinejad A, Hormazdi M (2005). Family history of colorectal cancer in Iran. BMC Cancer.

[39] Kurzawski G, Suchy J, Debniak T, Kladny J, Lubinski J (2004). Importance of microsatellite instability (MSI) in colorectal cancer: MSI as a diagnostic tool. Ann Oncol.

[40] Lee PY, Fletcher WS, Sullivan ES, Vetto JT (1994). Colorectal cancer in young patients: characteristics and outcome. Am Surg.

[41] Goshayeshi L, Khooiee A, Ghaffarzadegan K, Rahmani Khorram M, Bishehsari F, Hoseini B (2017). Screening for Lynch syndrome in cases with colorectal carcinoma from Mashhad. Arch Iran Med.

[42] Khorram MR, Goshayeshi L, Maghool F, Bergquist R, Ghaffarzadegan K, Eslami S, et al. Prevalence of mismatch repair-deficient colorectal adenoma/polyp in early-onset, advanced cases: a cross-sectional study based on Iranian hereditary colorectal Cancer registry. J Gastrointest Cancer. 2020. 10.1007/s12029-020-00395-y Epub 2020/03/21. PubMed PMID: 32193764. 10.1007/s12029-020-00395-y 32193764

[43] Lagerstedt Robinson K, Liu T, Vandrovcova J, Halvarsson B, Clendenning M, Frebourg T, et al. Lynch syndrome (hereditary nonpolyposis colorectal cancer) diagnostics. J Natl Cancer Inst. 2007;99(4):291–9. Epub 2007/02/22. 10.1093/jnci/djk051. PubMed PMID: 17312306. 10.1093/jnci/djk051 17312306

[44] Vasen HF, Watson P, Mecklin JP, Lynch HT (1999). New clinical criteria for hereditary nonpolyposis colorectal cancer (HNPCC, Lynch syndrome) proposed by the international collaborative group on HNPCC. Gastroenterology.

[45] Umar A, Boland CR, Terdiman JP, Syngal S, de la Chapelle A, Rüschoff J (2004). Revised Bethesda Guidelines for hereditary nonpolyposis colorectal cancer (Lynch syndrome) and microsatellite instability. J Natl Cancer Inst.

[46] Jacobs D, Zhu R, Luo J, Grisotti G, Heller DR, Kurbatov V (2018). Defining early-onset colon and rectal cancers. Front Oncol.

[47] Sudarshan V, Hussain N, Gahine R, Mourya J (2013). Colorectal cancer in young adults in a tertiary care hospital in Chhattisgarh. Raipur Indian J Cancer.

[48] Aykan NF, Yalçın S, Turhal NS, Özdoğan M, Demir G, Özkan M (2015). Epidemiology of colorectal cancer in Turkey: a cross-sectional disease registry study (a Turkish oncology group trial). Turk J Gastroenterol.

[49] Ansari R, Mahdavinia M, Sadjadi A, Nouraie M, Kamangar F, Bishehsari F (2006). Incidence and age distribution of colorectal cancer in Iran: results of a population-based cancer registry. Cancer Lett.

[50] Malekzadeh R, Bishehsari F, Mahdavinia M, Ansari R (2009). Epidemiology and molecular genetics of colorectal cancer in Iran: a review. Arch Iran Med.

[51] Mousavi SM, Gouya MM, Ramazani R, Davanlou M, Hajsadeghi N, Seddighi Z (2009). Cancer incidence and mortality in Iran. Ann Oncol.

[52] Fazeli MS, Adel MG, Lebaschi AH (2007). Colorectal carcinoma: a retrospective, descriptive study of age, gender, subsite, stage, and differentiation in Iran from 1995 to 2001 as observed in Tehran University. Dis Colon Rectum.

[53] Azadeh S, Moghimi-Dehkordi B, Fatem SR, Pourhoseingholi MA, Ghiasi S, Zali MR (2008). Colorectal cancer in Iran: an epidemiological study. Asian Pac J Cancer Prev.

[54] Siegel R, Desantis C, Jemal A (2014). Colorectal cancer statistics, 2014. CA Cancer J Clin.

[55] Sung JJ, Lau JY, Goh KL, Leung WK (2005). Increasing incidence of colorectal cancer in Asia: implications for screening. Lancet Oncol.

[56] Ashktorab H, Vilmenay K, Brim H, Laiyemo AO, Kibreab A, Nouraie M (2016). Colorectal cancer in young african americans: is it time to revisit guidelines and prevention?. Dig Dis Sci.

[57] Siraj AK, Prabhakaran S, Bavi P, Bu R, Beg S, Hazmi MA (2015). Prevalence of Lynch syndrome in a middle eastern population with colorectal cancer. Cancer.

[58] Alqahtani M, Grieu F, Carrello A, Amanuel B, Mashour M, Alattas R (2016). Screening for Lynch syndrome in young colorectal Cancer patients from Saudi Arabia using microsatellite instability as the initial test. Asian Pac J Cancer Prev.

[59] Parramore JB, Wei JP, Yeh KA (1998). Colorectal cancer in patients under forty: presentation and outcome. Am Surg.

[60] Schofield L, Watson N, Grieu F, Li WQ, Zeps N, Harvey J (2009). Population-based detection of Lynch syndrome in young colorectal cancer patients using microsatellite instability as the initial test. Int J Cancer.

[61] Minardi AJ, Sittig KM, Zibari GB, McDonald JC (1998). Colorectal cancer in the young patient. Am Surg.

[62] Chiang JM, Chen MC, Changchien CR, Chen JS, Tang R, Wang JY (2003). Favorable influence of age on tumor characteristics of sporadic colorectal adenocarcinoma: patients 30 years of age or younger may be a distinct patient group. Dis Colon Rectum.

[63] Palmer ML, Herrera L, Petrelli NJ (1991). Colorectal adenocarcinoma in patients less than 40 years of age. Dis Colon Rectum.

[64] Fante R, Benatti P, di Gregorio C, De Pietri S, Pedroni M, Tamassia MG (1997). Colorectal carcinoma in different age groups: a population-based investigation. Am J Gastroenterol.

[65] Makela JT, Kiviniemi H (2010). Clinicopathological features of colorectal cancer in patients under 40 years of age. Int J Color Dis.

[66] Endreseth BH, Romundstad P, Myrvold HE, Hestvik UE, Bjerkeset T, Wibe A (2006). Rectal cancer in the young patient. Dis Colon Rectum.

[67] Moore PA, Dilawari RA, Fidler WJ (1984). Adenocarcinoma of the colon and rectum in patients less than 40 years of age. Am Surg.

[68] Leff DR, Chen A, Roberts D, Grant K, Western C, Windsor AC (2007). Colorectal cancer in the young patient. Am Surg.

[69] Eker B, Ozaslan E, Karaca H, Berk V, Bozkurt O, Inanc M (2015). Factors affecting prognosis in metastatic colorectal cancer patients. Asian Pac J Cancer Prev.

[70] Firouraghi N, Bagheri N, Kiani F, Goshayeshi L, Ghayour-Mobarhan M, Kimiafar K (2020). A spatial database of colorectal cancer patients and potential nutritional risk factors in an urban area in the Middle East. BMC Res Notes.

[71] Ali Dadashi, Alireza Mohammadi, Shahab MohammadEbrahimi, Robert Bergquist, Ali Shamsoddini, Azam Hesami, Elahe Pishgar, Khalil Kimiafar, Masoumeh Sarbaz & Behzad Kiani (2021) ‎Spatial analysis of the 10 most prevalent cancers in north-eastern Iran, 2017–2018‎, Journal of Spatial Science. 10.1080/14498596.2021.1975583.

[72] Kiani B, Amin FH, Bagheri N, Bergquist R, Mohammadi AA, Yousefi M (2021). Association between heavy metals and colon cancer: an ecological study based on geographical information systems in north-eastern Iran. BMC Cancer.

[73] Chen J, Etzel CJ, Amos CI, Zhang Q, Viscofsky N, Lindor NM (2009). Genetic variants in the cell cycle control pathways contribute to early onset colorectal cancer in Lynch syndrome. Cancer Causes Control.

[74] Yaghoubi N, Soltani A, Ghazvini K, Hassanian SM, Hashemy SI (2019). PD-1/ PD-L1 blockade as a novel treatment for colorectal cancer. Biomed Pharmacother.

[75] Parry S, Win AK, Parry B, Macrae FA, Gurrin LC, Church JM (2011). Metachronous colorectal cancer risk for mismatch repair gene mutation carriers: the advantage of more extensive colon surgery. Gut.

[76] South CD, Hampel H, Comeras I, Westman JA, Frankel WL, de la Chapelle A (2008). The frequency of Muir-Torre syndrome among Lynch syndrome families. J Natl Cancer Inst.

