# Supplementary material for: Colorectal Cancer in North-Eastern Iran: a retrospective, comparative study of early-onset and late-onset cases based on data from the Iranian hereditary colorectal cancer registry
Source: BMC Cancer. 2022 Jan 8;22:48. doi: 10.1186/s12885-021-09132-5 (PMC8742430; doi:10.1186/s12885-021-09132-5)
Supplement: Supplementary file 1 — Additional file 1. [file 12885_2021_9132_MOESM1_ESM.docx]

**ANNEX**

**Amsterdam II criteria**

At least three relatives with an HNPCC-associated cancer (cancer of the colo-rectum, endometrium, small bowel, ureter or renal pelvis)

- One should be a first-degree relative to the other two;

- At least two successive generations should be affected;

- At least one should be diagnosed before age 50;

- Familial adenomatous polyposis should be excluded; and

- Tumors should be verified by pathological examination.

**Reference**

Vasen HF, Watson P, Mecklin JP, Lynch HT. New clinical criteria for hereditary non-polyposis colorectal cancer (HNPCC, Lynch Syndrome) proposed by the International Collaborative Group on HNPCC. Gastroenterology. 1999;116:1453-1456.

**Revised Bethesda guidelines**

- Individual with CRC diagnosed by age 50

- Individual with synchronous or metachronous CRC, or other HNPCC-associated tumors regardless of age

- Individual with CRC and MSI-H histology diagnosed by age 60

- Individual with CRC and more than 1 FDR with an HNPCC-associated tumour, with one cancer diagnosed by age 50

- Individual with CRC and more than 2 FDRs or SDRs with an HNPCC-associated tumour, regardless of age

**Reference**

Umar A, Boland CR, Terdiman JP, Syngal S, de la Chapelle, A. et al. Revised Bethesda Guidelines for hereditary non- polyposis colorectal cancer (Lynch Syndrome) and microsatellite instability. J Natl Cancer Inst. 2004; 96: 261-268.
